# The potential therapeutic effect of melatonin in gastro-esophageal reflux disease

**DOI:** 10.1186/1471-230X-10-7

**Published:** 2010-01-18

**Authors:** Tharwat S Kandil, Amany A Mousa, Ahmed A El-Gendy, Amr M Abbas

**Affiliations:** 1 Gastro-Enterology Surgical Center, Faculty of Medicine, Mansoura University, Mansoura, Egypt; 2 Internal Medicine Department, Specialized Medical Hospital, Faculty of Medicine, Mansoura University, Mansoura, Egypt; 3 Physiology Department, Faculty of Medicine, Mansoura University, Mansoura, Egypt

## Abstract

**Background:**

Gastro-Esophageal Reflux Disease (GERD) defined as a condition that develops when the reflux of stomach contents causes troublesome symptoms and/or complications. Many drugs are used for the treatment of GERD such as omeprazole (a proton pump inhibitor) which is a widely used antiulcer drug demonstrated to protect against esophageal mucosal injury. Melatonin has been found to protect the gastrointestinal mucosa from oxidative damage caused by reactive oxygen species in different experimental ulcer models. The aim of this study is to evaluate the role of exogenous melatonin in the treatment of reflux disease in humans either alone or in combination with omeprazole therapy.

**Methods:**

36 persons were divided into 4 groups (control subjects, patients with reflux disease treated with melatonin alone, omeprazole alone and a combination of melatonin and omeprazole for 4 and 8 weeks) Each group consisted of 9 persons. Persons were subjected to thorough history taking, clinical examination, and investigations including laboratory, endoscopic, record of esophageal motility, pH-metry, basal acid output and serum gastrin.

**Results:**

Melatonin has a role in the improvement of Gastro-esophageal reflux disease when used alone or in combination with omeprazole. Meanwhile, omeprazole alone is better used in the treatment of GERD than melatonin alone.

**Conclusion:**

The present study showed that oral melatonin is a promising therapeutic agent for the treatment of GERD. It is an effective line of treatment in relieving epigastric pain and heartburn. However, further studies are required to confirm the efficacy and long-term safety of melatonin before being recommended for routine clinical use.

**Trial Registration:**

QA13NCT00915616

## Background

Gastro esophageal reflux Disease (GERD) is defined as a condition that develops when the reflux of stomach contents causes troublesome symptoms and/or complications [1]. Symptoms of GERD occur in approximately 14 to 20% of the population on at least a weekly basis. Symptoms of GERD may result in a large burden on employers through increased absenteeism and decreased performance while remaining at work impaired by health problems [2].

Even though over 50 years have passed since the discovery of melatonin, the knowledge on its physiological function is still not complete. The results of the researches have provided the evidence that melatonin is synthesized not only in the pineal gland, but also in different organs. A special attention has been directed to the digestive tract where total quantity of melatonin is considerably greater than in the pineal gland [3]. It was calculated that the gastrointestinal tract contains at least 400 times melatonin than the pineal gland [4]. Although pineal melatonin acts prevalently in an endocrine capacity, extra-pineal melatonin may act as an autocrine or a paracrine hormone [5]. It protects gastric mucosa against destructive activity of free radicals in stress-induced ulcers and due to non-steroidal anti-inflammatory drugs and other gastrotoxic agents [6]. Furthermore, Kato et al. [7] demonstrated the inhibitory action of melatonin on secretion of HCL and pepsin. Only a few studies on gastrointestinal role of melatonin have been carried out on humans.

In view of melatonin's known gut regulatory functions, antidepressant and anxiolytic properties, and its potentially beneficial effects on brain gut axis, it was hypothesized that melatonin might serve as an effective agent for treating GERD.

## Methods

Sixty subjects "60", suffering from GERD symptoms, were attending the outpatient clinics of the Gastro-Enterology Center and Specialized Medical Hospital. Informed consent was obtained from all patients to be included in the study, after explanations of the nature of the disease and treatment options. All patients signed an informed consent to be included in our study. **This paper was approved by the local ethical committee of Mansoura University Hospital, General Surgery Department**.

All individuals were subjected to thorough history taking including: history of drug intake, epigastric pain, upper gastrointestinal bleeding (hematemesis and/or melena), dyspeptic manifestations and heartburn. They were also subjected to clinical examination with special stress on: (a) General examination: pulse, blood pressure and temperature. (b) Chest and heart examination. (c) Local abdominal examination for the state of the liver, spleen and the presence or absence of ascites. They also underwent **laboratory investigations**: Complete blood picture, Urine and stool analysis and Liver function tests including serum albumin, bilirubin, alanine transaminase enzyme (ALT), aspartate transaminase enzyme (AST). The patients excluded from our study were; patients with cardiac disease, patients with renal affection, and patients with liver diseases (drug induced, autoimmune disease and viral hepatitis). In addition, patients on the drugs known to affect the GIT motility (phenothiazines, anticholinergics, nitrates or calcium channel blockers) excluded during the time of conduction of the study or the preceding two weeks. After exclusion, 45 subjects were selected for evaluation by: a) Endoscopic Investigation: subjects were subjected to upper gastrointestinal endoscopy for visualization of the mucosa of esophagus and stomach for the presence of mucosal ulceration. b) Record of esophageal motility by Smart graph analysis software lab motility system Sandhill 8 - channels esophageal manometry. Manometric recording systems rely on computers for data acquisition display and analysis. This manometric recording was done by using station pull through technique at 5 minutes intervals. The most important aspect of Lower Esophageal Sphincter (LES) pressure measurement is that of LES relaxation. Subjects with duodenal, gastric ulcers or functional dyspepsia were excluded and finally 27 subjects with GERD were selected to complete the study compared with nine healthy volunteers who matched with age and sex as controls or reference.

After admission the following was done: (a) blood samples for measuring serum melatonin levels taken at 10.00 p.m., 2.00 a.m. and 6.00 a.m. After collecting the sample, the blood centrifuged and serum thus was obtained and frozen at the temperature of minus 80°C. Melatonin concentration was measured with ELISA method using the Lab system Multiscan and antibodies of the Immuno-Biological Laboratories (catalogue RE 54021) [8]. (b)An ambulatory digitrapper was used to perform 24 hours pH-metry measurement by UP S2020 Orion MMS Holland pH-metry. The pH probe was positioned 5 cm above the position of the LES. The data were collected using De-meester (DM) score; gastroesophageal reflux considered as a drop in esophageal PH below "4" and the percentage reflux in 24 hours calculated for each patient [9].

### Basal Acid Output (BAO)

At the end of each pH-metry monitoring period, the electrode was removed and BAO was measured during one hour through a naso-gastric tube inserted at the most dependent part of the stomach. The correct position of the tube was checked by the water recovery test. Patients were laid in a semi-recumbent position on their left side. Gastric secretion was aspirated continuously by gentle manual suction and collected in 15-minute samples after discarding the first 15-minute collection corresponding to the emptying of the stomach. The concentration of H^+ ^ions was determined on each sample by a titrimetric method with NaOH 0.1N and expressed in millimoles H^+ ^per litre. BAO is the sum of the four 15-minute outputs and is expressed in mmol H^+^/h.

### Determination of serum gastrin levels

The serum gastrin concentration was measured by a radioimmunoassay method using C-terminal-directed antibodies equally reactive to little gastrins (G-17) and big gastrins G-34. Serum Gastrin was expressed in picograms per milliliter.

Groups: The subjects selected were classified into four groups: Group I included nine healthy normal subjects and was considered the control group. Group II included nine patients suffering from GERD; receiving melatonin alone for treatment of GERD in a dose of 3 mg once daily at the bedtime [10]. Group III included nine patients suffering from GERD, receiving omeprazole alone for treatment of GERD in a dose of 20 mg twice daily [11]. Group IV included nine patients suffering from GERD receiving omeprazole and melatonin for treatment of GERD in the same dose of each of them. The three patient groups were re-evaluated after 4 weeks and 8 weeks of treatment.

### Statistical Analysis

The statistical analysis was performed using the SPSS statistical Package version 13.0 (SPSS, Chicago, IL, USA). To compare the data, the recorded values were expressed as means and standard deviation (Mean ± SD). The minimal level of significance was identified at p < 0.05 [12].

## Results

Table 1 illustrates that pretreated Patients with GERD showed heartburn, epigastric pain, and significant decrease in LES pressure, residual pressure, relaxation percentage, pH, serum gastrin and mean melatonin level and a significant increase in relaxation duration and basal acid output (BAO) relative to control group.

**Table 1:** Comparison of control subjects and patients pretreated with melatonin, omeprazole and both (n = 9)

	control	Pretreatment with melatonin	Pretreatment with omeprazole	Pretreatment with melatonin and omeprazole
**Symptoms:**				
**1-Heart burn:**				
**Yes**	0	7	7	8
**Duration(months)**		1.3 ± 0.4	1.2 ± 0.3	1.4 ± 0.4

**2-Epigastric pain:**				
**Yes**	0	6	6	6
**Duration(months)**		1.4 ± 0.5	1.3 ± 0.4	1.3 ± 0.4

**A)LES Study:**				
**1-LES pressure(mmHg)**	22.8 ± 1.3	10 ± 1.58^a^	10.5 ± 2.86^a^	10.3 ± 1.68^a^

**2-Residual pressure (mmHg)**	0.4 ± 0.01	0.012 ± 0.52^a^	0.1 ± 0.064^a^	0.012 ± 0.44^a^

**3-Relaxation duration (seconds)**	5.0 ± 0.1	6.8 ± 0.12^a^	6.5 ± 2.74^a^	6.8 ± 0.16^a^

**4-Relaxation %**	100 ± 0.00	86 ± 0.87^a^	87.2 ± 0.17^a^	85 ± 1.58^a^

**C) PH (at 5 cm above the LES):**	7.8 ± 0.4	2.3 ± 0.36^a^	2.1 ± 0.38^a^	1.98 ± 0.37^a^

**D) BAO **(mmol/h)	2.6 ± 0.6	24.7 ± 0.5^a^	25.1 ± 0.6^a^	24.9 ± 0.7^a^

**E) Serum Gastrin**(pg/ml)	41.8 ± 7.1	22.1 ± 4.2^a^	21.5 ± 4.6^a^	21.9 ± 4.7^a^

**D) Melatonin level at day time (pg/ml):**	36.1 ± 2.3	18.2 ± 5.54^a^	18.5 ± 3.75^a^	18.3 ± 3.8^a^

a: p < 0.05 relative to control

Table 2 illustrates that treatment with melatonin for 4 and 8 weeks leads to marked improvement of GERD symptoms regarding heartburn and epigastric pain. There was also a significant increase in the tone of the LES in the form of increased LES pressure, a significant increase in the residual pressure, a significant decrease in the relaxation duration, a significant increase in the relaxation percentage, a significant increase in the pH with a significant decrease in BAO, and a significant increase in serum gastrin and mean melatonin levels relative to pretreated patients.

**Table 2:** Effects of melatonin on patients with GERD group II (n = 9)

	Pretreatment with melatonin	4 weeks Post treatment with melatonin	8 weeks Post treatment with melatonin
**Symptoms:**			
**1-Heart burn:**			
**Yes**	7	3 (57.1%)	0 (100%)
**Duration(months)**	1.3 ± 0.4		

**2-Epigastric pain:**			
**Yes**	6	3 (50%)	1 (83%)
**Duration(months)**	1.4 ± 0.5		

**B)LES Study:**			
**1-LES pressure(mmHg)**	10 ± 1.58	14.5 ± 1.58^a^	20.2 ± 1.56 ^ab^

**2-Residual pressure (mmHg)**	0.012 ± 0.52	0.2 ± 0.016	0.32 ± 0.013^a^

**3-Relaxation duration (seconds)**	6.8 ± 0.12	5.9 ± 0.16 ^a^	5.3 ± 0.12 ^a^

**4-Relaxation %**	86 ± 0.87	90 ± 0.86 ^a^	100 ± 0.00 ^ab^

**C) PH (at 5 cm above the LES):**	2.3 ± 0.36	5.2 ± 0.5 ^a^	6.7 ± 0.65 ^ab^

**D) BAO **(mmol/h)	24.7 ± 0.5	20.1 ± 0.4 ^a^	16.6 ± 0.6 ^ab^

**E) Serum Gastrin**(pg/ml)	22.1 ± 3.2	27.2 ± 2.3 ^a^	32.3 ± 2.1 ^ab^

**D) Melatonin level at day time (pg/ml):**	18.2 ± 5.54	28.26 ± 2.26 ^a^	34.5 ± 2.35 ^ab^

a: p < 0.05 relative to pretreatment with melatonin

b: p < 0.05 relative to 4 weeks post treatment with melatonin

Table 3 illustrates that treatment with omeprazole for 4 and 8 weeks leads to marked improvement of GERD symptoms regarding heartburn and epigastric pain. There was also a non-significant change in the tone of the LES, a non-significant change in the residual pressure, relaxation duration, relaxation percentage, a significant increase in the pH with a significant decrease in BAO, a significant increase in serum gastrin and a non - significant increase in the melatonin level.

**Table 3:** Effects of omeprazole on patients with GERD Group III (n = 9)

	Pretreatment with omeprazole	4 weeks Post treatment with omeprazole	8 weeks Post treatment with omeprazole
**A) Symptoms:**			
**1-Heart burn:**			
**Yes**	7	2 (71.4%)	0 (100%)
**Duration(months)**	1.2 ± 0.3		

**2-Epigastric pain:**			
**Yes**	6	2	0
**Duration(months)**	1.3 ± 0.4	(66.7%)	(100%)

**B)LES Study:**			
**1-LES pressure(mmHg)**	10.5 ± 2.86	10.4 ± 4.05	10.5 ± 2.85

**2-Residual pressure (mmHg)**	0.1 ± 0.064	0.2 ± 0.15	0.1 ± 0.07

**3-Relaxation duration (seconds)**	6.5 ± 2.74	6.3 ± 2.7	6.3 ± 2.65

**4-Relaxation %**	87.2 ± 0.17	87.3 ± 0.25	87.1 ± 0.1

**C) PH (at 5 cm above the LES):**	2.1 ± 0.38	5.9 ± 0.48^a^	7.2 ± 0.32^ab^

**D) BAO **(mmol/h)	25.1 ± 0.6	17.2 ± 0.7^a^	11.5 ± 0.6^ab^

**E) Serum Gastrin **(pg/ml)	21.5 ± 4.6	32.1 ± 2.1^a^	35.9 ± 1.8^ab^

**D) Melatonin level at day time (pg/ml):**	18.5 ± 3.75	19.2 ± 3.47	17.9 ± 3.72

a: p < 0.05 relative to pretreatment with melatonin

b: p < 0.05 relative to 4 weeks Post treatment with melatonin

Table 4 illustrates that treatment with melatonin and omeprazole for 4 and 8 weeks leads to marked improvement of GERD symptoms regarding heartburn and epigastric pain. A significant increase in the tone of the LES in the form of increased LES pressure also noted. In addition, there is a significant increase in the residual pressure, a significant decrease in the relaxation duration, a significant increase in the relaxation percentage, a significant increase in the pH with a significant decreased in BAO and a significant increase in serum gastrin and melatonin levels.

**Table 4:** Effects of melatonin and omeprazole on patients with GERD group IV (n = 9)

	Pretreatment with melatonin and omeprazole	4 weeks Post treatment with melatonin and omeprazole	Post 8 weeks treatment with melatonin and omeprazole
**A) Symptoms:**			
**1-Heart burn:**			
**Yes**	8	1(87.5%)	0 (100%)
**Duration(months)**	1.4 ± 0.4		

**2-Epigastric pain:**			
**Yes**	6	1(83.3%)	0 (100%)
**ration(months)**	1.3 ± 0.4		

**B) LES Study:**			
**1-LES pressure(mmHg)**	10.3 ± 1.68	14.5 ± 1.26^a^	20.5 ± 1.22^ab^

**2-Residual pressure (mmHg)**	0.012 ± 0.44	0.21 ± 0.016	0.33 ± 0.016^a^

**3-Relaxation duration (seconds)**	6.8 ± 0.16	5.8 ± 0.13^a^	5.2 ± 0.12^a^

**4-Relaxation %**	85 ± 1.58	90 ± 1.23^a^	100 ± 0.00^ab^

**C) PH (at 5 cm above the LES):**	1.98 ± 0.37	6.1 ± 0.55^a^	7.5 ± 0.31^ab^

**D) BAO **(mmol/h)	24.9 ± 0.7	15.8 ± 0.9^a^	10.2 ± 0.9^ab^

**E) Serum Gastrin **(pg/ml)	21.9 ± 4.7	33.6 ± 2.7^a^	36.8 ± 2.1^ab^

**D) Melatonin level at day time (pg/ml):**	18.3 ± 3.8	28.83 ± 1.82^a^	34.5 ± 2.35^ab^

a: p < 0.05 relative to pretreatment with melatonin

b: p < 0.05 relative to 4 weeks Post treatment with melatonin

Table 5 illustrates that treatment with melatonin alone or combined with omeprazole (Group II & IV) leads to a significant increase in the tone of the LES in the form of increased LES pressure. Also, there is a significant increase in the residual pressure, a significant decrease in the relaxation duration, a significant increase in the relaxation percentage compared with patients treated with omeprazole alone (group III), while patients treated with omeprazole alone or combined with melatonin (group III & IV) showed a significant increase in PH and serum gastrin level with a significant decrease in BAO than melatonin alone (group II)

**Table 5:** Comparison of the three treated groups with GERD

	Melatonin	Omeprazole	Melatonin and Omeprazole	P value		
				
				P1	P2	P3
**A)Symptoms:**						
**Heart burn:**						
control	0	0	0			
pretreatment:						
yes	7	7	8			
duration (months)	1.3 ± 0.4	1.2 ± 0.3	1.4 ± 0.4			
4 weeks						
8 weeks	3(57.1%)	2(71.4%)	1(87.5%)			
	0(100%)	0(100%)	0(100%)			

**Epigastric pain:**						
-control	0	0	0			
pretreatment:						
yes	6	6	6			
duration (months)	1.4 ± 0.5	1.3 ± 0.4	1.3 ± 0.4			
4 weeks	3(50%)	2(66.7%)	1(83.3%)			
8 weeks	1(83%)	0(100%)	0(100%)			

**B) LES Study:**				P1	P2	P3
**1-LES pressure(mmHg)**						
control	22.8 ± 1.3	22.8 ± 1.3	22.8 ± 1.3	0.9	0.9	0.9
pretreatment:	10 ± 1.58	10.5 ± 2.86	10.3 ± 1.68	0.65	0.7	0.86
4 weeks	14.5 ± 1.58	10.4 ± 4.05	14.5 ± 1.26	0.006	0.468	0.002
8 weeks	20.2 ± 1.56	10.5 ± 2.85	20.5 ± 1.22	0.000	0.68	0.000

**2-Residual pressure (mmHg)**				P1	P2	P3
- control	0.4 ± 0.01	0.4 ± 0.01	0.4 ± 0.01	1.0	1.0	1.0
pretreatment:	0.012 ± 0.52	0.1 ± 0.064	0.012 ± 0.44	0.578	1.00	0.556
4 weeks	0.2 ± 0.016	0.2 ± 0.15	0.21 ± 0.016	1.00	0.198	0.198
8 weeks	0.32 ± 0.013	0.1 ± 0.07	0.33 ± 0.016	0.000	0.08	0.000

**3-Relaxation duration (seconds)**				P1	P2	P3
control	5.0 ± 0.1	5.0 ± 0.1	5.0 ± 0.1	1.0	1.0	1.0
pretreatment::	6.8 ± 0.12	6.5 ± 2.74	6.8 ± 0.16	0.747	1.000	0.747
4 weeks	5.9 ± 0.16	6.3 ± 2.7	5.8 ± 0.13	0.662	0.153	0.585
8 weeks	5.3 ± 0.12	6.3 ± 2.65	5.2 ± 0.12	0.747	0.102	0.059

**4-Relaxation %**				P1	P2	P3
control	100 ± 0.00	100 ± 0.00	100 ± 0.00	1.0	1.0	1.0
pretreatment:	86 ± 0.87	87.2 ± 0.17	85 ± 1.58	0.12	0.116	0.14
4 weeks	90 ± 0.86	87.3 ± 0.25	90 ± 1.23	0.1	1.000	0.1
8 weeks	100 ± 0.00	87.1 ± 0.1	100 ± 0.00	0.01	1.00	0.01

**C) PH (at 5 cm above the LES):**				P1	P2	P3
control:	7.8 ± 0.4	7.8 ± 0.4	7.8 ± 0.4	1.0	1.0	1.0
pretreatment:	2.3 ± 0.36	2.1 ± 0.38	1.98 ± 0.37	0.1	0.1	0.1
4 weeks	5.2 ± 0.5	5.9 ± 0.48	6.1 ± 0.55	0.008	0.002	0.09
8 weeks	6.7 ± 0.65	7.2 ± 0.32	7.5 ± 0.31	0.01	0.008	0.1

**D) BAO**(mmol/h)				P1	P2	P3
- control:	2.6 ± 0.6	2.6 ± 0.6	2.6 ± 0.6	1.0	1.0	1.0
pretreatment:	24.7 ± 0.5	25.1 ± 0.6	24.9 ± 0.7	0.1	0.1	0.1
4 weeks	20.1 ± 0.4	17.2 ± 0.7	15.8 ± 0.9	0.008	0.002	0.09
- 8 weeks	16.6 ± 0.6	11.5 ± 0.6	10.2 ± 0.9	0.01	0.008	0.1

**E) Serum Gastrin(pg/ml)**				P1	P2	P3
control:	41.8 ± 7.1	41.8 ± 7.1	41.8 ± 7.1	1.0	1.0	1.0
pretreatment:	22.1 ± 4.2	21.5 ± 4.6	21.9 ± 4.7	0.1	0.1	0.1
4 weeks	27.2 ± 2.3	32.1 ± 2.1	33.6 ± 2.7	0.008	0.002	0.09
- 8 weeks	32.3 ± 2.1	35.9 ± 1.8	36.8 ± 2.1	0.01	0.008	0.1

**D) Melatonin level at day time (pg/ml):**				P1	P2	P3
control	36.1 ± 2.3	36.1 ± 2.3	36.1 ± 2.3	1.0	1.0	1.0
pretreatment:	18.2 ± 5.54	18.5 ± 3.75	18.3 ± 3.8	0.1	0.665	0.472
4 weeks	28.26 ± 2.26	19.2 ± 3.47	28.83 ± 1.82	0.000	0.55	0.000
8 weeks	34.5 ± .35	17.9 ± 3.72	34.5 ± 2.35	0.000	1.00	0.000

P1: group III versus group II

P2: group IV versus group II

P3: group IV versus group III

## Discussion

Melatonin, a close derivative of serotonin (5-hydroxytryptamine, 5-HT) [13], is a hormone initiating sleep in humans [14] and a powerful scavenger of free radicals. It is more effective than several well-known vitamins [15]. The pineal gland is the major source of melatonin in the peripheral circulation, producing melatonin in a distinct circadian fashion, with peak levels occurring during the night [13]. Melatonin has been also detected in entero-endocrine (EE) cells of gastro-intestinal tract (GIT) wall, where this indole may act via endocrine, paracrine and/or luminal pathway through G-protein coupled receptors [16]. Following pinealectomy, the light/dark cycle of plasma melatonin levels disappears, while its daytime blood concentrations are attenuated but sustained mainly due to its release from the GIT, and therefore, a part of blood melatonin has a source in the digestive system, especially during daytime [17].

In the present study, the mean melatonin level was significantly lower in all patients in comparison to control group at baseline evaluation. This observation is in agreement with Klupiñska et al [18] who found that in patients with GERD and recurrent duodenal ulcers, melatonin concentration was lower than in healthy subjects and concluded that high or relatively correct secretion of melatonin is sufficient to prevent peptic changes in esophageal and duodenal mucosa. In addition, Bubenik et al. [19] demonstrated that pigs with chronic gastric ulcers exhibited lower contents of melatonin in the gastric mucosa and in the blood suggesting that these spontaneous ulcers originate from the local deficiency of the indole.

However, Otsuka et al. [20] reported that acute stress induced gastric lesions in rats are accompanied by increased plasma melatonin. The proposed explanation for the rise in melatonin is that its production increases under stressful stimuli in both, experimental animals and human as suggested by Karasek and Winczyk [21].

For the clinical purposes, some investigators measure serum concentration of melatonin twice, that is at 9.00 a.m. (light period) and 2.00 a.m. (dark period) and the differences in the day/night patterns taken for evaluation [22]. The interests of gstroenterologists mainly focused on studies on melatonin nocturnal secretion. In other investigations, melatonin concentration in blood measured at three points of time: at 10.00 p.m., 2.00 a.m. and 6.00 a.m. as done in our study as during these hours the influence of food intake on enterohormones secretion is negligible. On the other hand, at bedtime the patients often complain of recurrent symptoms of GERD that largely disturb their sleep [17]. It was also noted that pharmacologically administered low and high doses of melatonin have been found to be with very low or no toxicity [23].

The current study also evaluates the role of the recently used antiulcer drug melatonin, the widely used antiulcer drug omeprazole and the combination of both drugs in the treatment of GERD. We used an oral fast release melatonin at a dose of 3 mg/day for 4 and 8 weeks. Werbach [24] found that melatonin up to 6 mg at bedtime may be an effective treatment for GERD with fewer and less serious adverse effects. It was found that treatment of GERD with melatonin, omeprazole or both was duration dependent. Patients treated with melatonin for four weeks and patients treated with omeprazole for four weeks showed incomplete improvement of GERD symptoms. These findings are in agreement with Gavert and Harvey [25]. Moreover, in patients treated with melatonin for eight weeks and patients treated with melatonin and omeprazole for four weeks, there was complete improvement of GERD symptoms as heartburn and epigastric pain. These findings were in agreement with Pereira [26] who reported that dietary supplementation containing melatonin and L-tryptophan, which is a substrate for melatonin biosynthesis in patients with GERD, resulted in remarkable remission of GERD symptoms in the majority of treated patients. The clinical remission of GERD was comparable with that obtained by classical treatment using omeprazole. It was concluded that the formulation containing melatonin or its precursor, tryptophan, promotes regression of GERD symptoms without any side effects and may be useful in the GERD therapy. Melatonin has also been studied in alleviating GERD. In a head-to-head comparison, the researchers gave 175 patients standard treatment with the prescription drug omeprazole, while 176 received a supplement containing melatonin, its precursor L-tryptophan, and B vitamins, over a 40-day treatment period. All patients in the supplement group reported complete regression of symptoms by the end of the study, compared with only 66% in the drug-treated group. Again, no significant side effects were reported in the supplemented patients [26].

Although many studies were carried out to evaluate the role of melatonin in GERD based on its effect in alleviation of GERD symptoms only, the review of literature showed that no previous studies were based on endoscopic findings besides the clinical improvement.

In the present study, also signs were improved including lower esophageal sphincter (LES) pressure (from 10 ± 1.58 and, 10.3 ± 1.68 mmHg in the pretreated patients to 16.5 ± 0.6, and 14.1 ± 0.5 mmHg in patients treated with melatonin for 8 weeks and patients treated with melatonin and omeprazole for 4 weeks respectively). In addition, residual pressure (from 0.012 ± 0.52, and, 0.012 ± 0.44 in the pretreated patients to 0.32 ± 0.013, and, 0.21 ± 0.016 mmHg patients treated with melatonin for 8 weeks and patients treated with melatonin and omeprazole for 4 weeks respectively). Besides, relaxation duration (from 6.8 ± 0.12, and 6.8 ± 0.16 seconds in the pretreated patients to 5.3 ± 0.12, and 5.8 ± 0.13 seconds in patients treated with melatonin for 8 weeks and patients treated with melatonin and omeprazole for 4 weeks respectively), relaxation % (from 86 ± 0.87, and 85 ± 1.58% in pretreated patients to 95 ± 0.9 and 90 ± 1.23% in patients treated with melatonin for 8 weeks and patients treated with melatonin and omeprazole for 4 weeks respectively). However, the pH, 5 cm above the level of LES, was elevated from 2.3 ± 0.36, 2.1 ± 0.38 and 1.98 ± 0.37 in pretreated patients to 5.9 ± 0.65, 5.9 ± 0.48 and 6.1 ± 0.55 in patients treated with melatonin for 8 weeks, patients treated with omeprazole for 4 weeks and patients treated with melatonin and omeprazole for 4 weeks respectively).

It was believed that melatonin protects against GERD by increasing blood flow and anti-inflammatory molecules in the esophageal mucous, thus preventing significant esophageal injury [27]. Although, Sener-Muratoglu et al. [28] previously compared the antiulcer and gastro duodenal protective mechanism of famotidine, omeprazole and melatonin and their results revealed that the three drugs have gastroduodenal protective action but famotidine and omeprazole have lowering effects on gastric acidity (antisecrotory activity) whereas melatonin has no effect on this parameter but famotidine and omeprazole were not efficient as antioxidant as melatonin.

However, others concluded that the esophagoprotective activity of melatonin against GERD might be related to the inhibitory effect of this indole on gastric acid secretion and due to stimulation of gastrin release, which might attenuate the gastro-esophageal reflux by stimulation of the contractile activity of the lower esophageal sphincter [29].

In the present study, there was a significant increase in the pH with a significant decrease in BAO and a significant increase in serum gastrin after melatonin therapy compared to pretreatment levels. While patients treated with omeprazole alone or combined with melatonin showed a significant increase in PH and serum gastrin level with a significant decrease in BAO than melatonin alone, however, the combined therapy showed a non-significant increase in serum gastrin or BAO compared with omeprazole alone.

These protective effects were accompanied by gradual increase in plasma melatonin levels suggesting that topical melatonin exerts a local protective action on gastric mucosa, acting via circulation following its absorption form the gut. The results of our study revealed that melatonin has a role in the improvement of GERD as detected in patients treated with melatonin for 8 weeks and in patients treated with melatonin and omeprazole for 4 weeks. Meanwhile, omeprazole alone has a better effect on gastric acidity than melatonin alone as the clinical improvement started at the fourth week of treatment with omeprazole and was completed at the eighth week. On the other hand, with melatonin alone the improvement started at the fourth week and was completed at the eighth week of treatment but it was significantly less effective than omeprazole. It was found that melatonin accelerates the clinical and endoscopic improvement when combined with omeprazole as improvement in combination therapy was completed at shorter duration (at the fourth week) with no further increase in serum gastrin level. Our results were in agreement with Bandyopadhyay et al. [30] who stated that melatonin prevented gastric damage and when compared with already marketed anti-ulcer drugs such as ranitidine and omeprazole, melatonin was found to be more effective than ranitidine but less effective than omeprazole in preventing stress ulcer. They also demonstrated that co-treatment of GERD with melatonin at a low dose synergistically increases the efficacy of omeprazole in preventing stress induced lesion. This may be important, as giving omeprazole at lower doses would reduce the severity of their side effects. Rieter et al. [31] reported that melatonin when combined with other anti ulcer drugs like omeprazole has a beneficial effect as it accelerates the healing effects of omeprazole and shortens the duration of treatment. Therefore, melatonin reduces the side effects and increases the efficacy of omeprazole.

## Conclusion

From the results of our study, it can be concluded that melatonin could be used in the treatment of GERD either alone or in combination with omeprazole. The combination therapy of both melatonin and omeprazole is preferable as melatonin accelerates the healing effect of omeprazole and therefore shortens the duration of treatment and minimizes its side effects.

## Abbreviations

LES: lower esophageal sphincter; EC: enterochromaffin cells; NOS: Nitric Oxide Synthase.

## Competing interests

The authors declare that they have no competing interests.

## Authors' contributions

The manuscript has been seen and approved by all authors. TSK & AAM **write the paper**. TSK & AE **designed research**. TSK, AAM, AE & AMA **performed research**. TSK & AMA **analyzed data**.

## Pre-publication history

The pre-publication history for this paper can be accessed here:

http://www.biomedcentral.com/1471-230X/10/7/prepub
